# Safety of Cholecystectomy in Nonagenarians: A Systematic Review and Meta-Analysis

**DOI:** 10.3390/geriatrics11030069

**Published:** 2026-06-08

**Authors:** Dimitrios Vouros, Belen Conde Inarejos, James Fowler, Sarthak Jain, Vasileios Kotsarinis, Maaz Ullah, Awais Asif Chaudhary, Martyn Charles Stott, Ahmad Hassan, Shahin Hajibandeh, Shahab Hajibandeh, Jacob Kadamapuzha, Thomas Satyadas

**Affiliations:** 1 Department of Hepatobiliary and Pancreatic Surgery, Manchester Royal Infirmary Hospital, Manchester M13 9WL, UK; dimitrios.vouros@mft.nhs.uk (D.V.); belen.conde@mft.nhs.uk (B.C.I.); james.fowler@mft.nhs.uk (J.F.); sarthak.jain@mft.nhs.uk (S.J.); vasileios.kotsarinis@mft.nhs.uk (V.K.); maaz6097@gmail.com (M.U.); awais.chaudhary@mft.nhs.uk (A.A.C.); martyncharles.stott@mft.nhs.uk (M.C.S.); ahmedhassan@doctors.net.uk (A.H.); jacob.kadamapuzha@mft.nhs.uk (J.K.); thomas.satyadas@mft.nhs.uk (T.S.); 2 Department of Hepatobiliary and Pancreatic Surgery, Queen Elizabeth Hospital, Birmingham B15 2GW, UK; shahin.hajibandeh@uhb.nhs.uk; 3 Faculty of Medicine, Health and Life Science, Swansea University, Swansea SA2 8PP, UK

**Keywords:** cholecystectomy, nonagenarians, mortality, morbidity, laparoscopy, robotic, open, emergency, elective, conversion, meta-analysis, meta-regression

## Abstract

**Background/Objectives**: To evaluate the safety of cholecystectomy in nonagenarians. **Methods**: In compliance with the PRISMA statement standards, a systematic review including random-effects meta-analysis and meta-regression models was conducted. All studies reporting postoperative outcomes in patients aged ≥90 undergoing cholecystectomy were included and analyzed. **Results**: Six studies (1223 patients) were included. The risk of 30-day mortality was 5.4% (95% CI 3.1–7.7); 30-day morbidity occurred in 22% (95% CI 11.3–32.8). The mean length of hospital stay was 11.5 days (95% CI 8.3–14.6). Postoperative mortality was not affected by male sex (coefficient: 0.028, *p* = 0.832), ASA status ≥ III (coefficient: 0.051, *p* = 0.309), cholecystitis as indication for cholecystectomy (coefficient: −0.166, *p* = 0.051), cholecystectomy in emergency setting (coefficient: −0.020, *p* = 0.425), laparoscopic (coefficient: −0.104, *p* = 0.09) or open approach (coefficient: 0.104, *p* = 0.09), and conversion to open surgery (coefficient: 0.043, *p* = 0.820). The GRADE certainty of evidence was low to moderate. **Conclusions**: Subject to selection bias and confounding by fitness, the available evidence suggests that cholecystectomy in highly selected nonagenarians with good performance status, who have passed robust preoperative fitness assessment tests, may be safe with an acceptable risk of morbidity and mortality.

## 1. Introduction

Advances in living conditions, hygiene, medicine, and public health strategies resulted in impressive increase in the number of individuals aged above 90 [1,2]. It is estimated that nonagenarians will constitute approximately 71.16 million of the world population in 2050 [1]. In parallel, the number of nonagenarians and centenarians presenting with medical conditions indicating further medical investigations or interventions is growing.

Cholecystectomy is considered as high volume low complexity operation. Symptomatic gallstone disease is the most common indication for cholecystectomy. The incidence of gallstone disease increases with age; 24% of men and 35% of women will have gallstones by the age of 90 years [3]. Therefore, it is not uncommon to encounter a patient aged 90 or over with an indication for elective or emergency cholecystectomy. It has been shown that the likelihood of a patient undergoing a cholecystectomy for symptomatic gallstone disease decreases with age [4]. Recent studies investigated the outcomes of cholecystectomy in nonagenarians which encouraged us to complete a systematic review to evaluate safety of cholecystectomy in nonagenarians.

## 2. Materials and Methods

### 2.1. Compliance with Reporting and Methodological Standards

The protocol for the study was registered in a publicly available database of prospectively registered systematic reviews (PROSPERO registration number: CRD420261277483). The design of the study followed the Cochrane Handbook for Systematic Reviews (version 6.4) [5] and the reporting of the study followed the Preferred Reporting Items for Systematic reviews and Meta-Analyses (PRISMA) 2020 statement standards [6] (Appendix A). There was no deviation from the registered protocol.

### 2.2. Eligibility Criteria for Study Selection

The eligibility criteria are listed below:

- Population: All patients age ≥ 90 with an indication for cholecystectomy were considered eligible for inclusion
- Intervention: Cholecystectomy performed using any approach (laparoscopic, robotic, or open) in an elective or emergency setting was the intervention of interest.
- Comparison: The study had no comparison group
- Outcomes: Mortality with 30 days of operation was the primary outcome. The secondary outcomes included 30-day morbidity and length of hospital stay.
- Study design: All randomised controlled trials, cohort studies (prospective and retrospective), and case series (prospective and retrospective) with sample size ≥ 15 patients were considered for inclusion. The following articles were excluded: opinions, letters, case reports, correspondences, case-control studies, review articles, systematic reviews and meta-analyses.

### 2.3. Search Strategy

Two independent authors (D.V. and B.C.) searched Scopus, CENTRAL, MEDLINE, the ICTRP registry, the ISRCTN registry and ClinicalTrials.gov using a search strategy (with no language restrictions) including the following keywords: (nonagenarian [MeSH Terms] OR nonagenarian OR 90 years OR 90) AND (cholecystectomy [MeSH Terms] OR cholecystectomy). The last search was done on 20 November 2025. Moreover, in order to identify more eligible studies, the reference lists of potentially eligible articles were evaluated.

### 2.4. Screening and Selection of the Eligible Articles

The above eligibility criteria were used by two independent authors (V.K. and M.U.) to screen and select eligible articles. Firstly, the articles were screened by their title and abstracts. Then, irrelevant articles were excluded without full-text review. The full-text of relevant articles were assessed in detail to include the eligible studies. eligibility criteria were included. A third independent author (M.S.) resolved any disagreement in the findings of the two authors.

### 2.5. Data Variables and Data Collection

The data collection sheet included the following information: the name of first author, year in which the study was published, country in which the study was conducted, the journal in which the article was published, study design, sample size, age of the included patients, sex of the included patients, American Society of Anesthesiologists (ASA) status ≥ 3, cholecystitis as indication, emergency setting, elective setting, operative approach, conversion to open, and the outcomes. The above data variables were determined using pilot-testing technique by two independent authors (D.V. and B.C.) and disagreement was resolved by an independent author (S.H. (Shahab Hajibandeh)). The data were collected by two independent authors (J.F. and S.J.) and a third independent author resolved (J.K.) any disagreement in the findings of the two authors.

### 2.6. Study Risk of Bias and Evidence Certainty Assessment

The GRADE system was used to determine certainty of evidence and the ROBINS-I tool (The Risk Of Bias In Non-Randomized Studies of Interventions) was used by two independent authors (V.K. and M.U.) for risk of bias evaluation. A third independent author (M.S.) resolved any disagreement in the findings of the two authors.

### 2.7. Statistical Analyses

The OpenMeta [Analyst] software (version 1.0; Brown University, Providence, RI, USA) was used for analyses. Random-effects single-arm meta-analysis with an individual patient as the unit of analysis was performed to determine pooled outcomes with 95% confidence intervals (CIs). Forest plots with 95% confidence intervals (CIs) were constructed to present the results. The I^2^ using Cochran’s Q test (χ^2^) was determined to quantify statistical heterogeneity (I^2^ 0–25% means low heterogeneity; I^2^ 25–75% means moderate heterogeneity; I^2^ 75–100% means high heterogeneity). A multivariable meta-regression analysis was conducted including male sex, ASA status ≥ III, cholecystitis as indication for cholecystectomy, cholecystectomy in emergency setting, laparoscopic or open approach, and conversion to open surgery as independent variables and 30-day mortality as dependent variable. The results of meta-regression were presented in bubble plots. The meta-regression analyses were exploratory in nature aiming to explore potential confounders or associations rather than showing causation. The risk of publication bias could not be assessed using funnel plot because less than 10 studies were included.

### 2.8. Sensitivity Analyses

Sensitivity analyses included separate analysis of studies with low risk of bias and leave-one-out analysis.

## 3. Results

### 3.1. Search Results

**Figure 1:**
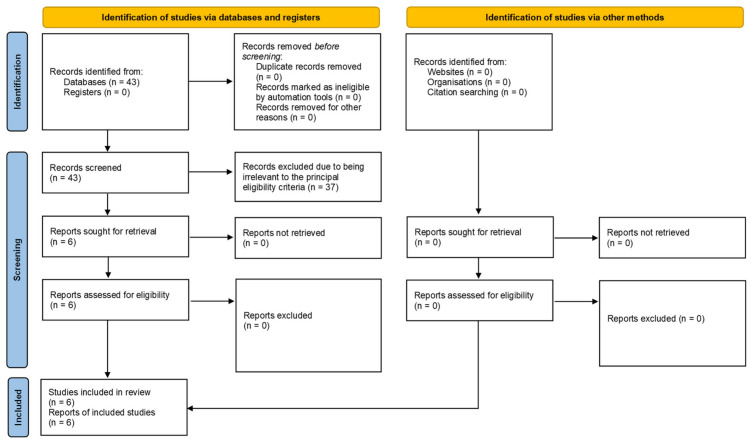
Study PRISMA flow diagram.

Figure 1 shows the PRISMA flow diagram. Forty-three articles were identified after searching the databases. Thirty-seven articles were irrelevant and were excluded without full-text review because they did not meet the principal eligibility criteria. No studies were excluded after full-text review. Therefore, six studies [7,8,9,10,11,12] including 1223 patients were found to be eligible after full-text review. Table 1 and Table 2 show the baseline characteristics of the included studies and the included population, respectively.

Ramírez-Giraldo 2023 [7] was a retrospective cohort study conducted in Colombia that included 102 patients aged ≥90 undergoing cholecystectomy. Lincango-Naranjo 2021 [8] was a retrospective cohort study conducted in Ecuador that included 15 patients aged ≥90 undergoing cholecystectomy. Kim 2021 [9] was a retrospective cohort study conducted in Korea that included 41 patients aged ≥90 undergoing cholecystectomy. Novello 2018 [10] was a retrospective cohort study conducted in Italy that included 36 patients aged ≥90 undergoing cholecystectomy. Irojah 2017 [11] was a retrospective cohort study conducted in USA that included 1007 patients aged ≥90 undergoing cholecystectomy. Dubecz 2012 [12] was a retrospective cohort study conducted in Germany that included 22 patients aged ≥90 undergoing cholecystectomy.

### 3.2. Study Risk of Bias Assessment

The risk of bias due to confounding and patient selection were judged to be high in all of the included studies. The risk of bias due to intervention classification and deviation from intended interventions were low in all six studies. Moreover, there was no concerns regarding missing data, outcome measurement and selective reporting in the included studies (Table 3).

### 3.3. Outcomes

#### 3.3.1. 30-Day Mortality

**Figure 2:**
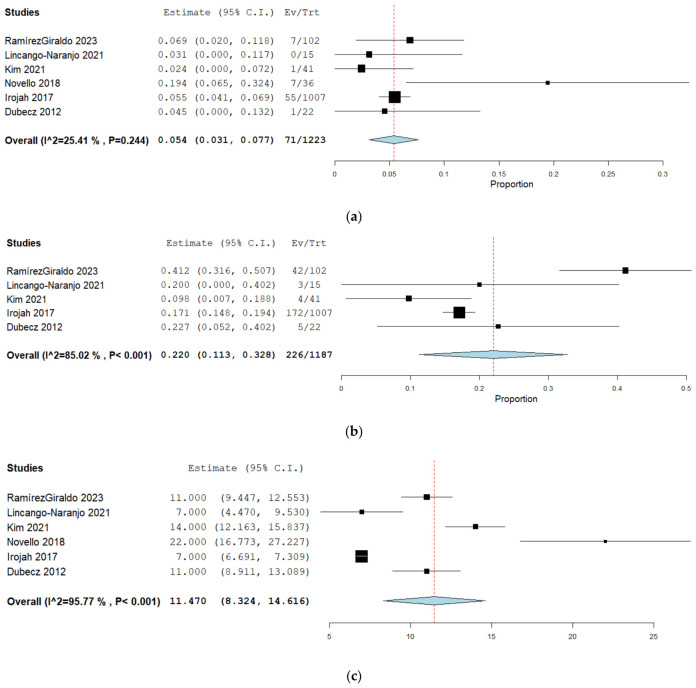
Forest plots for the outcomes after cholecystectomy in nonagenarians: (**a**) 30-day mortality; (**b**) 30-day morbidity; (**c**) length of hospital stay. Ramírez-Giraldo 2023 [7]; Lincango-Naranjo 2021 [8]; Kim 2021 [9]; Novello 2018 [10]; Irojah 2017 [11]; Dubecz et al. 2012 [12].

Analysis of 1223 patients from six studies showed that risk of 30-day mortality was 5.4% (95% CI 3.1–7.7) (Figure 2). The between-study statistical heterogeneity was moderate (I^2^ = 25%) and the GRADE certainty was moderate.

#### 3.3.2. 30-Day Morbidity

Analysis of 1187 patients from five studies showed that risk of 30-day morbidity was 22% (95% CI 11.3–32.8) (Figure 2). The between-study statistical heterogeneity was high (I^2^ = 85%) and the GRADE certainty was low.

#### 3.3.3. Length of Hospital Stay

Analysis of 1223 patients from six studies showed that the mean length of hospital stay was 11.5 days (95% CI 8.3–14.6) (Figure 2). The between-study statistical heterogeneity was high (I^2^ = 96%) and the GRADE certainty was low.

### 3.4. Meta-Regression Analysis

**Figure 3:**
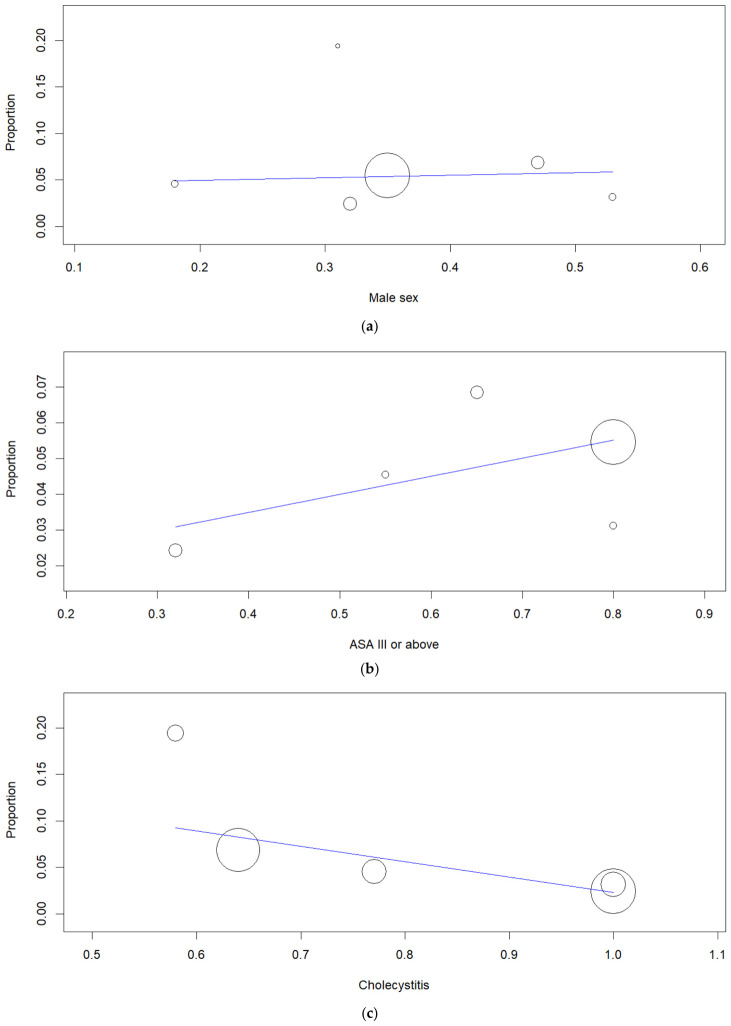

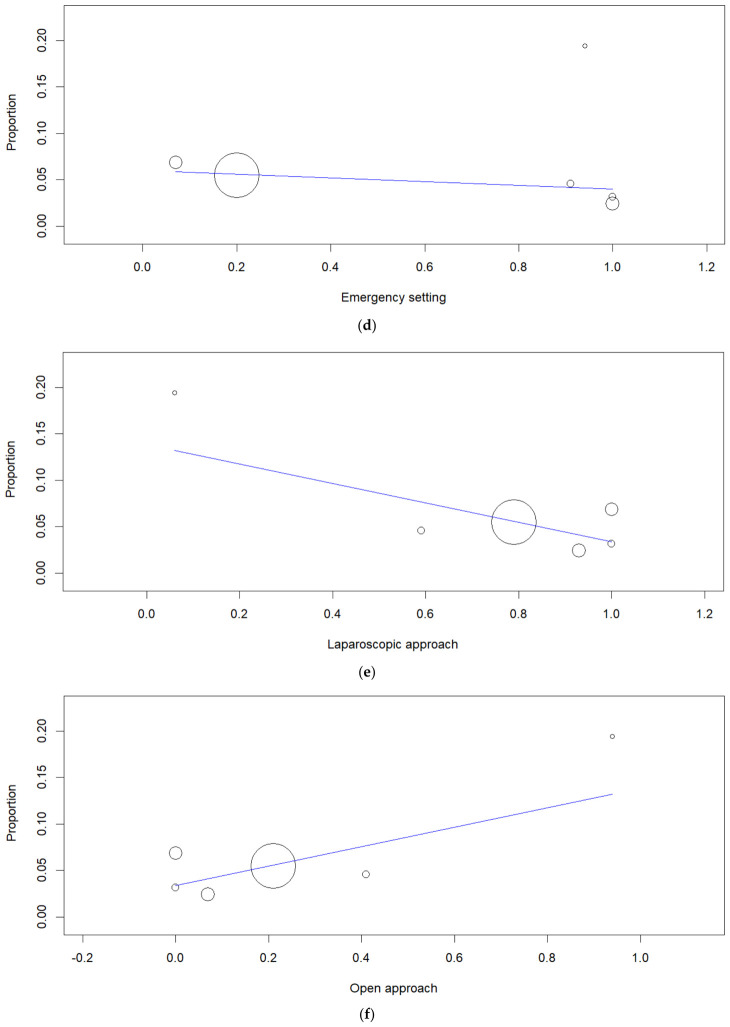

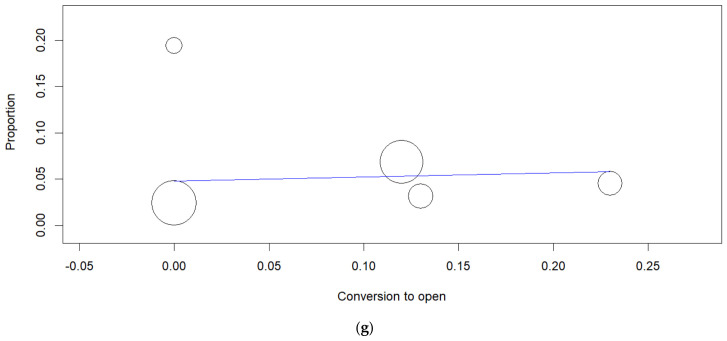
Bubble plots for the meta-regression analyses of postoperative mortality based on the following variables. (**a**) Male sex; (**b**) ASA status ≥ III; (**c**) Cholecystitis; (**d**) Emergency setting; (**e**) Laparoscopic approach; (**f**) Open approach; (**g**) Conversion to open.

Meta-regression analysis showed that postoperative mortality was not affected by male sex (coefficient: 0.028, *p* = 0.832), ASA status ≥ III (coefficient: 0.051, *p* = 0.309), cholecystitis as indication for cholecystectomy (coefficient: −0.166, *p* = 0.051), cholecystectomy in emergency setting (coefficient: −0.020, *p* = 0.425), laparoscopic (coefficient: −0.104, *p* = 0.09) or open approach (coefficient: 0.104, *p* = 0.09), and conversion to open surgery (coefficient: 0.043, *p* = 0.820) (Figure 3).

### 3.5. Sensitivity Analysis

**Figure 4:**
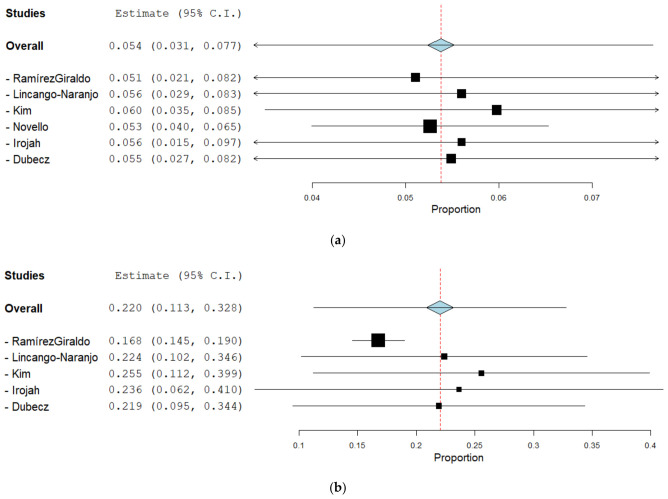

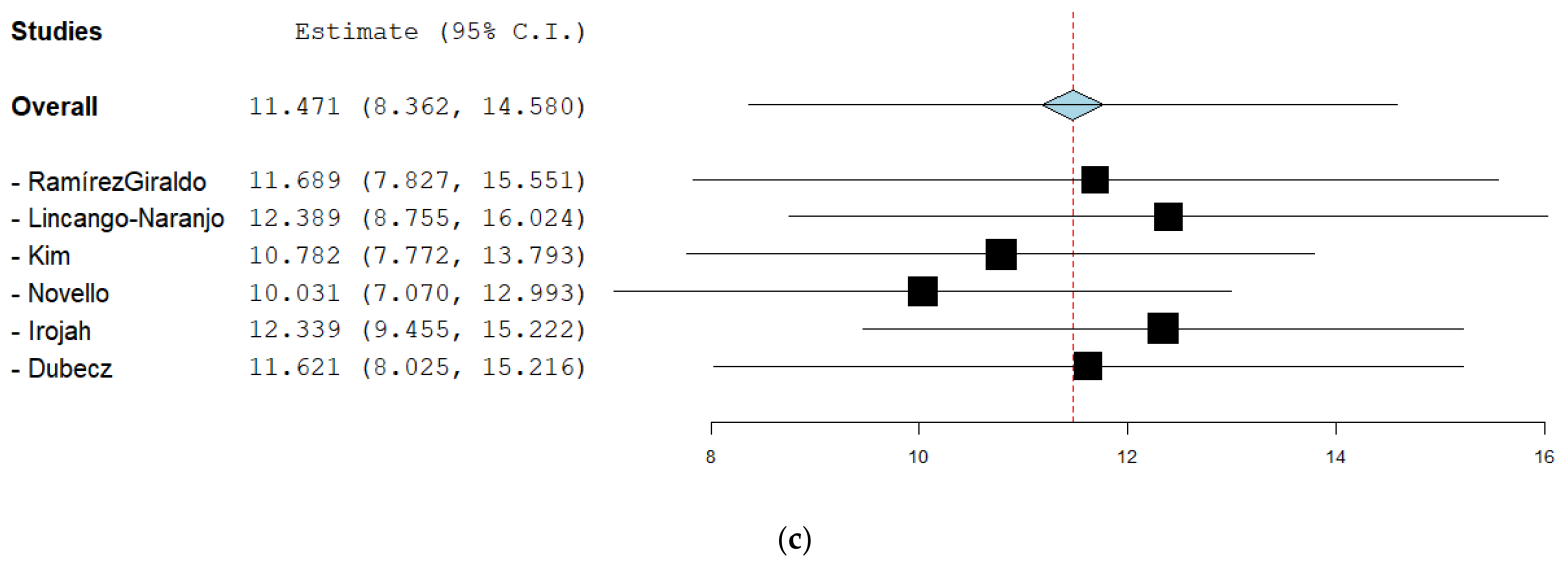
Results of leave-one-out analysis for: (**a**) 30-day mortality; (**b**) 30-day morbidity; (**c**) length of hospital stay. Ramírez-Giraldo 2023 [7]; Lincango-Naranjo 2021 [8]; Kim 2021 [9]; Novello 2018 [10]; Irojah 2017 [11]; Dubecz et al. 2012 [12].

Separate analyses of studies with low risk of bias and leave-one-out analysis showed consistency of the results (Figure 4). Sensitivity analyses identified the study by Ramírez-Giraldo et al. as source of heterogeneity for 30-day morbidity, the removal of which reduced I^2^ from 85% to 0% without affecting the overall morbidity rate (16.8%, 95% CI 14.5–19.0).

## 4. Discussion

The safety of cholecystectomy in nonagenarians was evaluated in this systematic review and meta-analysis, which analyzed 1223 patients from six studies. Based on the results of the analyses, cholecystectomy in nonagenarians was associated with acceptable risk of 30-day mortality (5.4%; moderate certainty) and morbidity (22%; low certainty). Postoperative mortality was not affected by male sex, ASA status ≥ III, cholecystitis as indication for cholecystectomy, cholecystectomy in emergency setting, laparoscopic or open approach, and conversion to open surgery.

The safety of cholecystectomy in nonagenarians has not been investigated in any previous systematic reviews; however, the results can be compared with studies conducted in a younger cohort of patients. Lluís et al. [13] conducted a retrospective study of 212 octogenarians who underwent cholecystectomy and concluded that cholecystectomy was associated with 1.5–13.2% risk of postoperative mortality and 26.5–48.5% of postoperative morbidity [13]. A recent meta-analysis showed that the risk of mortality after cholecystectomy in octogenarians was 2.8–3.3% [14]. In addition, Park et al. [15] demonstrated safety and feasibility of cholecystectomy in octogenarians [15]. Consequently, safety and feasibility of cholecystectomy in octogenarians may justify why it may be safe in selected cohort of nonagenarians.

In the study by Ramírez-Giraldo et al. [7] the risk of mortality was 6.8% in nonagenarians, 1.7% in patients aged 70–89, and 0% in younger patients. On the other hand, In the study by Novello et al. [10] the risk of mortality 19.4% in nonagenarians versus 3.1% in octogenarians. However, the risk of mortality was comparable between nonagenarians and octogenarians (2.4% vs. 2.3%) in the study by Kim et al. [9]. The results of these studies suggest that although the risk of mortality after cholecystectomy may be higher in nonagenarians compared with octogenarians, it may still be considered safe in selected nonagenarians.

The mean length of hospital stay was 11.5 days. This is significantly longer than the typical length of stay expected after cholecystectomy [16]. It has been shown that cholecystectomy in the elderly is associated with longer length of hospital stay compared with younger population [17]. This highlights that although performing cholecystectomy may be technically safe in selected nonagenarians, their postoperative recovery is likely to be complex, demanding a geriatrician-led multidisciplinary approach to prevent delirium, manage multiorgan vulnerabilities, and preserve functional independence [18]. Unfortunately, the available evidence on outcomes such as discharge destination (home versus nursing home) and quality of life is lacking and should be the subject of future research.

The recent evidence suggests that age on its own may not increase the risk of operative mortality and morbidity. Sarcopenia as objective measure of age-related physiological decline may be a better predictor of outcomes in patients undergoing abdominal surgery [19]. Therefore, it can be argued that any patients, regardless of age, who have good performance status with appropriate physiological reserve and muscle mass may be offered cholecystectomy in the presence of an indication for cholecystectomy.

Although the results of the current study suggest the safety of cholecystectomy in nonagenarians, it should be highlighted that the available evidence is derived from retrospective studies that operated on patients who were already fit candidates for cholecystectomy, and a large proportion of patients with indications for cholecystectomy who were considered high risk were not included in the included studies. Therefore, this selection bias highlights that cholecystectomy should be performed in a very select cohort of nonagenarians who are considered fit candidates for the operation based on an objective assessment of age-related physiological decline and vulnerability.

### 4.1. Limitations

This study has the following limitations. The risk of selection bias, confounding by indication, and confounding by fitness for operation were inevitable due to the retrospective design of the included studies. Approximately 82% (1007 out of 1223 patients) of the total patient population originated from a single study; although the sensitivity analyses showed that the results were independent of individual studies, this should be taken into account in terms of the generalizability of the findings and the risk of type 2 error in the other included studies with a smaller sample size. Moreover, the between-study heterogeneity was moderate to high. The heterogeneity was high for 30-day morbidity and length of hospital stay. Regarding 30-day morbidity, the sensitivity analysis identified the study by Ramírez-Giraldo et al. as source of heterogeneity. Removing it reduced heterogeneity without affecting the overall morbidity rate. The observed heterogeneity for length of hospital stay can be explained by the variations in indications for cholecystectomy, the operative approach used, institutional protocols, patient selection criteria, and the regional healthcare system. We conducted meta-regression analyses which showed that the results were not affected by the evaluated variables. However, the results of meta-regression should be interpreted with caution, as only six studies were included in the analyses and the results may be subject to type 2 error. The meta-regression analyses were exploratory in nature to explore potential confounders or associations; consequently, the results of meta-regression analyses should not be interpreted as causation. The current study did not include patients who underwent percutaneous cholecystostomy instead of cholecystectomy; therefore, whether the outcomes of patients undergoing cholecystostomy is comparable to those who underwent cholecystectomy remained unanswered. The current study lacks a comparison arm due to lack of comparative evidence; therefore, the outcomes could not be compared between the nonagenarians and younger patients. However, in absence of adequate comparative evidence, meta-analysis of single-arm studies may be considered as the best available evidence to quantify the outcomes and to provide rationale for future comparative studies. Finally, publication bias could not be assessed formally because the number of included studies was less than 10; therefore, selective reporting cannot be excluded.

### 4.2. Directions for Future Research

Based on the results of the current study and the acknowledged limitations, it may be reasonable to highlight the following directions for future research. The available evidence is confounded by fitness for surgery. The objective measures of fitness for surgery were not reported in the included studies. Therefore, future studies should specify the performance status of their patients and preoperative measures used to assess the patients’ fitness for surgery. The eligibility criteria in future studies should be based on the inclusion of surgically fit patients, and the results of the current study support the safety of cholecystectomy in such patients. Moreover, future studies should compare the outcomes between cholecystectomy and percutaneous cholecystostomy (as a less invasive procedure) in this cohort of patients. The outcomes of interest in future studies should include discharge destination (home versus nursing home), quality of life, and 90-day mortality.

## 5. Conclusions

Subject to selection bias and confounding by fitness, the available evidence suggests that cholecystectomy in highly selected nonagenarians with good performance status, who have passed robust preoperative fitness assessment tests, may be safe with an acceptable risk of morbidity and mortality. Future studies should investigate important outcomes, including discharge destination, quality of life, and 90-day mortality. Moreover, the outcomes of cholecystectomy versus percutaneous cholecystostomy in this cohort of patients should be the focus of future research.

## Figures and Tables

**Table 1:** Details of the included studies.

Study	Year	Country	Journal	Design	Included Patients	Sample Size
Ramírez-Giraldo et al. [7]	2023	Colombia	Surgical Endoscopy	Retrospective observational	Patients aged ≥90 undergoing cholecystectomy	102
Lincango-Naranjo et al. [8]	2021	Ecuador	Cirugía y cirujanos	Retrospective observational	Patients aged ≥90 undergoing cholecystectomy	15
Kim et al. [9]	2021	Korea	Journal of Minimally Invasive Surgery	Retrospective observational	Patients aged ≥90 undergoing cholecystectomy	41
Novello et al. [10]	2018	Italy	World Journal of Surgery	Retrospective observational	Patients aged ≥90 undergoing cholecystectomy	36
Irojah et al. [11]	2017	USA	Permanente journal	Retrospective observational	Patients aged ≥90 undergoing cholecystectomy	1007
Dubecz et al. [12]	2012	Germany	Journal of Gastrointestinal Surgery	Retrospective observational	Patients aged ≥90 undergoing cholecystectomy	22

**Table 2:** Details of the included patients.

Study	Male Sex	ASA III or Above	Cholecystitis as Indication for Cholecystectomy	Emergency Setting	Laparoscopic	Open	Conversion to Open
Ramírez-Giraldo et al. [7], 2023, Colombia	48/102	66/102	65/102	7/102	102/102	0/102	12/102
Lincango-Naranjo et al. [8], 2021, Ecuador	8/15	12/15	15/15	15/15	15/15	0/15	2/15
Kim et al. [9], 2021, Korea	13/41	13/41	41/41	34/41	38/41	3/41	0/38
Novello et al. [10], 2018, Italy	11/36	NR	21/36	34/36	2/36	34/36	0/36
Irojah et al. [11], 2017, USA	353/1007	805/1007	NR	200/1007	791/1007	216/1007	NR
Dubecz et al. [12], 2012, Germany	4/22	12/22	17/22	20/22	13/22	9/22	3/13

ASA: American Society of Anesthesiologists; NR: Not reported.

**Table 3:** Results of risk of bias assessment.

Study	ROBINS-I Risk of Bias Assessment						
	Confounding	Participants Selection	Intervention Classification	Deviations from Intended Interventions	Missing Data	Outcomes Measurement	Selective Reporting
Ramírez-Giraldo et al. [7]	High risk	High risk	Low risk	Low risk	Low risk	Low risk	Low risk
Lincango-Naranjo et al. [8]	High risk	High risk	Low risk	Low risk	Low risk	Low risk	Low risk
Kim et al. [9]	High risk	High risk	Low risk	Low risk	Low risk	Low risk	Low risk
Novello et al. [10]	High risk	High risk	Low risk	Low risk	Low risk	Low risk	Low risk
Irojah et al. [11]	High risk	High risk	Low risk	Low risk	Low risk	Low risk	Low risk
Dubecz et al. [12]	High risk	High risk	Low risk	Low risk	Low risk	Low risk	Low risk

ROBINS-I: The Risk Of Bias In Non- Randomized Studies of Interventions.

## Data Availability

The original contributions presented in this study are included in the article. Further inquiries can be directed to the corresponding author.

## Appendix A

**Table A1:** PRISMA 2020 Checklist.

Section and Topic	Item #	Checklist Item	Location Where Item is Reported
Title			
Title	1	Identify the report as a systematic review.	1
Abstract			
Abstract	2	See the PRISMA 2020 for Abstracts checklist.	1
Introduction			
Rationale	3	Describe the rationale for the review in the context of existing knowledge.	2
Objectives	4	Provide an explicit statement of the objective(s) or question(s) the review addresses.	2
Methods			
Eligibility criteria	5	Specify the inclusion and exclusion criteria for the review and how studies were grouped for the syntheses.	2
Information sources	6	Specify all databases, registers, websites, organisations, reference lists, and other sources searched or consulted to identify studies. Specify the date when each source was last searched or consulted.	2, 3
Search strategy	7	Present the full search strategies for all databases, registers, and websites, including any filters and limits used.	2, 3
Selection process	8	Specify the methods used to decide whether a study met the inclusion criteria of the review, including how many reviewers screened each record and each report retrieved, whether they worked independently, and if applicable, details of automation tools used in the process.	3
Data collection process	9	Specify the methods used to collect data from reports, including how many reviewers collected data from each report, whether they worked independently, any processes for obtaining or confirming data from study investigators, and if applicable, details of automation tools used in the process.	3
Data items	10a	List and define all outcomes for which data were sought. Specify whether all results that were compatible with each outcome domain in each study were sought (e.g., for all measures, time points, analyses), and if not, the methods used to decide which results to collect.	3
	10b	List and define all other variables for which data were sought (e.g., participant and intervention characteristics, funding sources). Describe any assumptions made about any missing or unclear information.	3
Study risk of bias assessment	11	Specify the methods used to assess risk of bias in the included studies, including details of the tool(s) used, how many reviewers assessed each study and whether they worked independently, and if applicable, details of automation tools used in the process.	3
Effect measures	12	Specify for each outcome the effect measure(s) (e.g., risk ratio, mean difference) used in the synthesis or presentation of results.	3
Synthesis methods	13a	Describe the processes used to decide which studies were eligible for each synthesis (e.g., tabulating the study intervention characteristics and comparing against the planned groups for each synthesis (item #5)).	3
	13b	Describe any methods required to prepare the data for presentation or synthesis, such as handling of missing summary statistics, or data conversions.	3
	13c	Describe any methods used to tabulate or visually display results of individual studies and syntheses.	3
	13d	Describe any methods used to synthesize results and provide a rationale for the choice(s). If meta-analysis was performed, describe the model(s), method(s) to identify the presence and extent of statistical heterogeneity, and software package(s) used.	3
	13e	Describe any methods used to explore possible causes of heterogeneity among study results (e.g., subgroup analysis, meta-regression).	3
	13f	Describe any sensitivity analyses conducted to assess robustness of the synthesized results.	3
Reporting bias assessment	14	Describe any methods used to assess risk of bias due to missing results in a synthesis (arising from reporting biases).	3
Certainty assessment	15	Describe any methods used to assess certainty (or confidence) in the body of evidence for an outcome.	3
Results			
Study selection	16a	Describe the results of the search and selection process, from the number of records identified in the search to the number of studies included in the review, ideally using a flow diagram.	4
	16b	Cite studies that might appear to meet the inclusion criteria, but which were excluded, and explain why they were excluded.	4
Study characteristics	17	Cite each included study and present its characteristics.	4, 5
Risk of bias in studies	18	Present assessments of risk of bias for each included study.	5, 6
Results of individual studies	19	For all outcomes, present, for each study: (a) summary statistics for each group (where appropriate) and (b) an effect estimate and its precision (e.g., confidence/credible interval), ideally using structured tables or plots.	6–10
Results of syntheses	20a	For each synthesis, briefly summarise the characteristics and risk of bias among contributing studies.	6–10
	20b	Present results of all statistical syntheses conducted. If meta-analysis was done, present for each the summary estimate and its precision (e.g., confidence/credible interval) and measures of statistical heterogeneity. If comparing groups, describe the direction of the effect.	6–10
	20c	Present results of all investigations of possible causes of heterogeneity among study results.	10
	20d	Present results of all sensitivity analyses conducted to assess the robustness of the synthesized results.	10
Reporting biases	21	Present assessments of risk of bias due to missing results (arising from reporting biases) for each synthesis assessed.	NA
Certainty of evidence	22	Present assessments of certainty (or confidence) in the body of evidence for each outcome assessed.	6, 7
Discussion			
Discussion	23a	Provide a general interpretation of the results in the context of other evidence.	10, 11
	23b	Discuss any limitations of the evidence included in the review.	11, 12
	23c	Discuss any limitations of the review processes used.	11, 12
	23d	Discuss implications of the results for practice, policy, and future research.	12
Other information			
Registration and protocol	24a	Provide registration information for the review, including register name and registration number, or state that the review was not registered.	2
	24b	Indicate where the review protocol can be accessed, or state that a protocol was not prepared.	2
	24c	Describe and explain any amendments to information provided at registration or in the protocol.	2
Support	25	Describe sources of financial or non-financial support for the review, and the role of the funders or sponsors in the review.	13
Competing interests	26	Declare any competing interests of review authors.	13
Availability of data, code and other materials	27	Report which of the following are publicly available and where they can be found: template data collection forms; data extracted from included studies; data used for all analyses; analytic code; any other materials used in the review.	13
